# High resolution imaging of human development: shedding light on contrast agents

**DOI:** 10.1007/s00234-024-03413-z

**Published:** 2024-07-12

**Authors:** Karl Jacobs, Daniel Docter, Lotte de Smit, Hans A. M. Korfage, Sophie C. Visser, Frank Lobbezoo, Ruslan Hlushchuk, Bernadette S. de Bakker

**Affiliations:** 1 Department of Orofacial Pain and Disfunction, Academic Centre for Dentistry Amsterdam (ACTA), University of Amsterdam and VU University Amsterdam, Amsterdam, The Netherlands; 2 Department of Medical Biology, Section Clinical Anatomy & Embryology, Amsterdam UMC location AMC, University of Amsterdam, Meibergdreef 15, Amsterdam, The Netherlands; 3 Amsterdam Reproduction and Development Research Institute, Meibergdreef 9, Amsterdam, The Netherlands; 4 Micro-CT Research Group, Institute of Anatomy, University of Bern, Baltzerstrasse 2, CH-3012 Bern, Switzerland; 5 Department of Obstetrics and Gynecology, Amsterdam UMC location AMC, University of Amsterdam, Amsterdam Reproduction & Development Research Institute, Meibergdreef 9, Amsterdam, The Netherlands; 6 Erasmus MC – Sophia Children’s Hospital, University Medical Center Rotterdam, Department of Pediatric Surgery, Rotterdam, The Netherlands

**Keywords:** Vascular development, Micro-CT, Contrast agents, Microvascular tissue imaging, High-resolution imaging techniques

## Abstract

**Background:**

Visualizing (micro)vascular structures remains challenging for researchers and clinicians due to limitations in traditional radiological imaging methods. Exploring the role of vascular development in craniofacial malformations in experimental settings can enhance understanding of these processes, with the effectiveness of high-resolution imaging techniques being crucial for successful research in this field. Micro-CT imaging offers 3D microstructural insights, but requires contrast-enhancing staining agents (CESAs) for visualizing (micro)-vascular tissues, known as contrast-enhanced micro-CT (CECT). As effective contrast agents are crucial for optimal visualization, this review focuses on comparative studies investigating such agents for micro-vascular tissue imaging using micro-CT. Furthermore, we demonstrate the utilization of B-Lugol solution as a promising contrast agent for acquiring high-quality micro-CT images of (micro)vascular structures in human embryonic samples.

**Method:**

This scoping review followed Preferred Reporting Items for Systematic Reviews and Meta-analysis Protocols. PubMed database provided relevant articles, screened initially by title and abstract. Inclusion and exclusion criteria defined outcomes of interest.

**Results:**

From an initial search, 273 records were identified, narrowed down to 9 articles after applying our criteria. Additionally, two articles were added through citation searching. This, a total of 11 articles were incorporated in this study.

**Conclusion:**

This micro-CT contrast agent review underscores the need for tailored choices based on research goals. Both Barium sulfate and Iodine-based agents showing excellent results, providing high resolution (micro) vascular content, especially in *ex-vivo* specimens. However, careful consideration of protocols and tissue characteristics remains imperative for optimizing the effectiveness of micro-CT imaging for the study of cranio-facial vascular development.

## Introduction

Blood vessels constitute the body's circulatory system, responsible for the transportation of blood, nutrients, and oxygen throughout the entire body. Vasculogenesis and angiogenesis, the formation and proliferation of (new) blood vessels, plays a vital role in health and disease, significantly impacting everyday life. Notably, angiogenesis is a key factor in the growth and progression of tumors and the development of cardiovascular diseases [11, 34]. Therapeutic angiogenesis holds great promise as a prospective treatment strategy. It offers the potential to modulate microcirculation, thereby providing a valuable avenue for aiding cardiovascular and cancer patients worldwide [17, 32]. As such, research in this domain is essential to advance our understanding and establish effective therapeutic interventions for angiogenesis-related conditions.

In addition to therapeutic angiogenesis research, there is significant value in exploring embryonic vascularization, particularly within the craniofacial region. Developmental malformations can be linked directly to cerebrovascular disorders, including stroke, aneurysms, and arteriovenous malformations [20, 30, 41]. Moreover, vascular development plays a pivotal role in the growth of all body structures. Concerning craniofacial development, various factors may be implicated in their etiology. These factors can be linked to genetic diseases, chromosomal mutations, or abnormal embryonic development. Congenital craniofacial deformities represent inherent anomalies that impact the bones of the skull and facial soft tissue defects, including craniosynostosis, craniofacial fissure, dilated orbital distance, and craniofacial microscopic anomalies. Many causal factors remain elusive, as these can be linked to various syndromes or might be present as isolated conditions [13]. The vascular network in the embryonic head and neck region is very complex. It is plausible that developmental craniofacial malformations are related to impaired angiogenesis [14, 31]). Therefore, extensive research is warranted to shed light on the angio- and vasculogenesis processes during craniofacial embryonic development. Such investigations provide valuable information on the potential relationship between angiogenesis and the development of craniofacial malformations.

A substantial body of research has already explored the field of angiogenesis, with most studies relying on animal models and exploring a variety of visualization techniques, including MRI, micro-CT, CT-scans, histology, and others. Numerous contrast agents are currently available, each with its unique application method and intended contrast objectives. This variety can pose challenges in identifying the most suitable contrast agents for imaging (micro)vascular structures. Micro-CT stands out as a valuable tool for visualizing embryonic vasculature in both *in-vivo* and *ex-vivo* samples, surpassing MRI in terms of cost and time efficiency while providing superior resolution [9, 21, 5]. In contrast to clinical CT scanners, which typically produce images with voxel sizes in the range of 1 mm, micro-CT has the capability to provide images with voxel sizes in the micrometer range [38]. Micro-CT imaging holds the capability for achieving high-resolution images of vascular tissue, offering non-destructive 3D microstructural information without the need for fixation or optical clearing. However, the effective utilization of micro-CT relies on careful consideration of contrast agents, as each option presents specific advantages and disadvantages [15, 22, 25]. For optimal visualization of soft tissues or vascular structures using absorption-based micro-CT, the introduction of a contrast-enhancing staining agent (CESA) is necessary. CESAs are chemical compounds incorporating X-ray attenuating elements into tissue to provide contrast in scans.

The aim of this scoping review was to comprehensively delineate the existing body of research pertaining to contrast agents used in vascular imaging. The primary objective was to identify potential gaps in knowledge, specifically focusing on the understanding of various contrast agents and their characteristics. Given the anticipation that limited research has been undertaken on the utilization of contrast agents in embryonic contexts, this study also encompassed articles that explored the application of contrast agents in small animal models. When aiming for visualization of early cranio-facial vascular development, intravascular contrast agents are unsuitable due to ongoing developmental processes and lack of vascular continuity. Immersion techniques, particularly utilizing buffered Lugol (B-Lugol), have been shown effective by Dawood et al. Additionally, to this literature search, we assessed the feasibility of B-Lugol’s solution to acquire high-resolution images from two embryos (gestational age 7 and 8,5 weeks) stained using the Tescan Unitom XL for imaging Background.

## Application methods

In addition to prioritizing image quality, resolution, and contrast, the suitability of the contrast agent also plays a crucial role in the search for optimal contrast agents. In this section, we provide context for understanding the diverse application techniques of contrast agents, emphasizing their utilization via intravascular and immersing methods, along with the distinct characteristics relevant for *in-vivo* and *ex-vivo* imaging applications.

### In-vivo application methods

In regard to *in-vivo* contrast agents, several application methods can be used: intravenous injection, intra-arterial injection, and cardiac injections techniques. When applying the intravenous injection technique, the contrast agent is directly introduced into the bloodstream through a catheter or tail vein injection in case of experimental animals. This approach allows real-time imaging of blood vessels within living organisms, capturing dynamic processes. However, careful consideration of the injection site and rate is essential for even distribution of the contrast agent.

Intra-arterial injection contrast agents are administered directly into specific arteries, providing high-resolution imaging of targeted vascular territories. This method may require more specialized techniques and can be more invasive compared to intravenous injection.

Intracardiac Injection, involves injecting contrast agents directly into the heart chambers, enabling comprehensive imaging of complex cardiac vascular structures. Precision is crucial to avoid damage to the heart and surrounding tissues.

The most commonly clinically employed contrast agents for *in-vivo* application methods predominantly consist of iodine-based materials for attenuation, such as Iomeprol (Imeron^®^) especially for X-ray and Iohexol (Omnipaque^®^) for Computed tomography (CT). Additionally, Barium Sulfate is also frequently chosen for this purpose (gastrointestinal, through swallowing).

### Ex-vivo application methods

In regard to *ex-vivo* contrast agents several application methods can be used; Perfusion Fixation, Immersion and Soaking and Vascular Casting. When applying the perfusion-based fixation, the vasculature is often pre-flushed with a PBS-Heparin combination and then flushed with a solidifying contrast medium followed by immersion of the sample in a fixative solution (for example, 2% paraformaldehyde solution in PBS) to preserve the structural integrity. This method offers detailed imaging of the entire vascular network [18].

When performing the immersion and soaking technique, samples are immersed or soaked in a contrast/staining agent solution. This straightforward method is particularly impactful with smaller sample sizes and is suitable for post-mortem investigations. Nonetheless, extended immersion durations may be necessary, particularly with larger samples, and the penetration of the contrast agent into tissues can fluctuate [7].

Another method utilized is vascular corrosion casting. This technique involves perfusion of the vascular tree with a solidifying material to produce vascular replica. Vascular casting effectively preserves the three-dimensional structure of the vasculature, but careful removal of surrounding tissues is necessary to expose the obtained cast. This is usually achieved through chemical or enzymatic maceration (corrosion) of the surrounding tissues. Vascular corrosion casts technique followed by scanning electron microscopy provides more 3D information that histological approaches and has been commonly used in the past. The major limitation is that due to corrosion technique the tissue surrounding the vessels as well as vessel walls themselves are completely removed, which impairs the detection of the location and mapping of the visualized vessels or further investigation of the morphology of the vessel wall [17].

In ex-vivo scenarios, iodine-based contrast agents like Lugol are frequently used, often with the option of buffering [4]. Additionally, Barium Sulfate is a commonly chosen alternative. Moreover, there are several other contrast agents available on the market for intravascular ex-vivo applications, including gold nanoparticles, Phosphotungstic acid (PTA), lead, and PU4ii@, a hydrophobic polyurethane-based resin.

## Research methods

### Protocol and registration

The aim of this scoping review was to comprehensively delineate the existing body of research pertaining to contrast agents used in vascular imaging. In this scoping review, our emphasis is on comparative literature, as opposed to case reports, which only offer specific details about individual contrast agents. Comparative literature reveals preferred contrast agents and highlights their differences. Additionally, to this literature search, we assessed the feasibility of B-Lugol’s solution to acquire high-resolution images from two embryos (gestational age 7 and 8,5 weeks) stained using the Tescan Unitom XL for imaging. This scoping review was established using the Preferred Reporting Items for Systematic Reviews and Meta-analysis Protocols (PRISMA) [40]. This study is performed according to the ethical guidelines at Academic Centre of Dentistry Amsterdam ACTA (protocol number 2023–14043).

### Information sources

PubMed was utilized for this query. Additional searches were conducted to find more articles of interest by examining the references cited in the included articles, based on the search strategy on PubMed.

### The following search-string was employed in Pubmed for the extraction of relevant articles.

(((("X-Ray Microtomography"[Mesh] OR microtomograph*[tiab] OR micro-tomograph*[tiab] OR "microcomputed tomograph*"[tiab] OR "micro-computed tomograph*"[tiab] OR micro-ct*[tiab] OR micro-cat*[tiab] OR microangioct*[tiab] OR microangio-ct*[tiab]) AND (vascular*[tiab] OR "blood vessel*"[tiab] OR cadaver*[tiab] OR "ex vivo"[tiab]))) AND ("Contrast Media"[Mesh] OR "contrast agent*"[tiab] OR "contrast medi*"[tiab])).

### Eligibility criteria

The inclusion criteria for the articles comprised studies that involved embryos or small animals as research subjects, while specifically focusing on the comparative investigations of contrast agents and their properties. Furthermore, articles were included in this review if they were written in English. Exclusion criteria were applied to studies in which an alternative device, distinct from micro-CT, was employed for investigating vascularization.

### Data items

We collected data from the included articles to provide a comprehensive overview of these studies, encompassing details about the specimen or animal model used, and about the specific contrast agents employed (Table 1). Detailed information about each contrast agent can be found in a separate section (Appendix 1). Additionally, we assessed the quality of high-resolution images obtained by scanning two embryos (at gestational ages 5 + 7 weeks and 8 + 6 weeks) stained with B-Lugol's solution using the Tescan Unitom XL.

**Table 1:** Summary of the characteristics of the articles included in this scoping review

Study	Country of origin	Test animal	Component of test animal	*n*	Comparison of contrast agents
Doost and Arnolda [8]	Australia	Mouse	Heart	60	Potassium Iodide vs. PTA
Dunmore-Buyze et al. [10]	Canada	Mouse	Heart	12	Iodine vs. PTA
Hong et al. [19]	USA	Mouse	Brain	24	Barium vs. Microfil^®^
Kingston et al. [22]	Australia	Rat	Lower limb	16	Barium sulfate vs. lead oxide
Le et al. [24]	Switzerland	Mouse	Whole body	2	PU4ii^®^ vs. XlinCA^®^
Liu et al. [26]	China	Rat	Cervical spinal cord	12	Barium sulfate vs. lead oxide
Lalwani et al. [23]	California	Mouse	Thorax	6	Fenestra VC^®^ vs. Isovue^®^ vs. ExiTron^®^
Nebuloni et al. [28]	Switzerland	Mouse	Head, neck and lower limb	44	Iomeprol vs. blood-pool agents
Roche et al. [33]	France	Mouse	Hind limb long bone	30	Barium vs. Microfil^®^
Schambach et al. [36]	Germany	Mouse	Whole body	26	Imeron^®^ vs. Fenestra VC^®^
Xu et al. [42]	China	Rabbit	Endplate*	20	Barium sulfate vs. Iohexol

### Synthesis of results

An analysis of the conclusions derived from the incorporated articles is conducted, and these findings are subsequently summarized in a tabular format (Table 2). These conclusions have been carefully considered to offer recommendations regarding the utilization of contrast agents in human embryos.

**Table 2:**
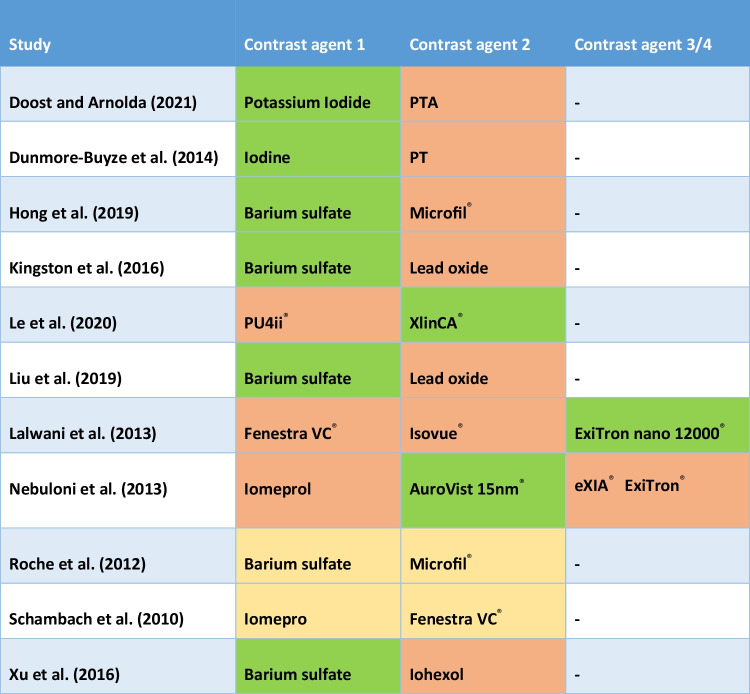
Outcomes of the comparisons of contrast agents included in this scoping review

Preferred contrast agent for visualizing vascular structures according to the article

Not identified as the optimal choice for visualizing vascular structures

No significant differences between the contrast agents in visualization of vascular structures

## Results

**Fig. 1:**
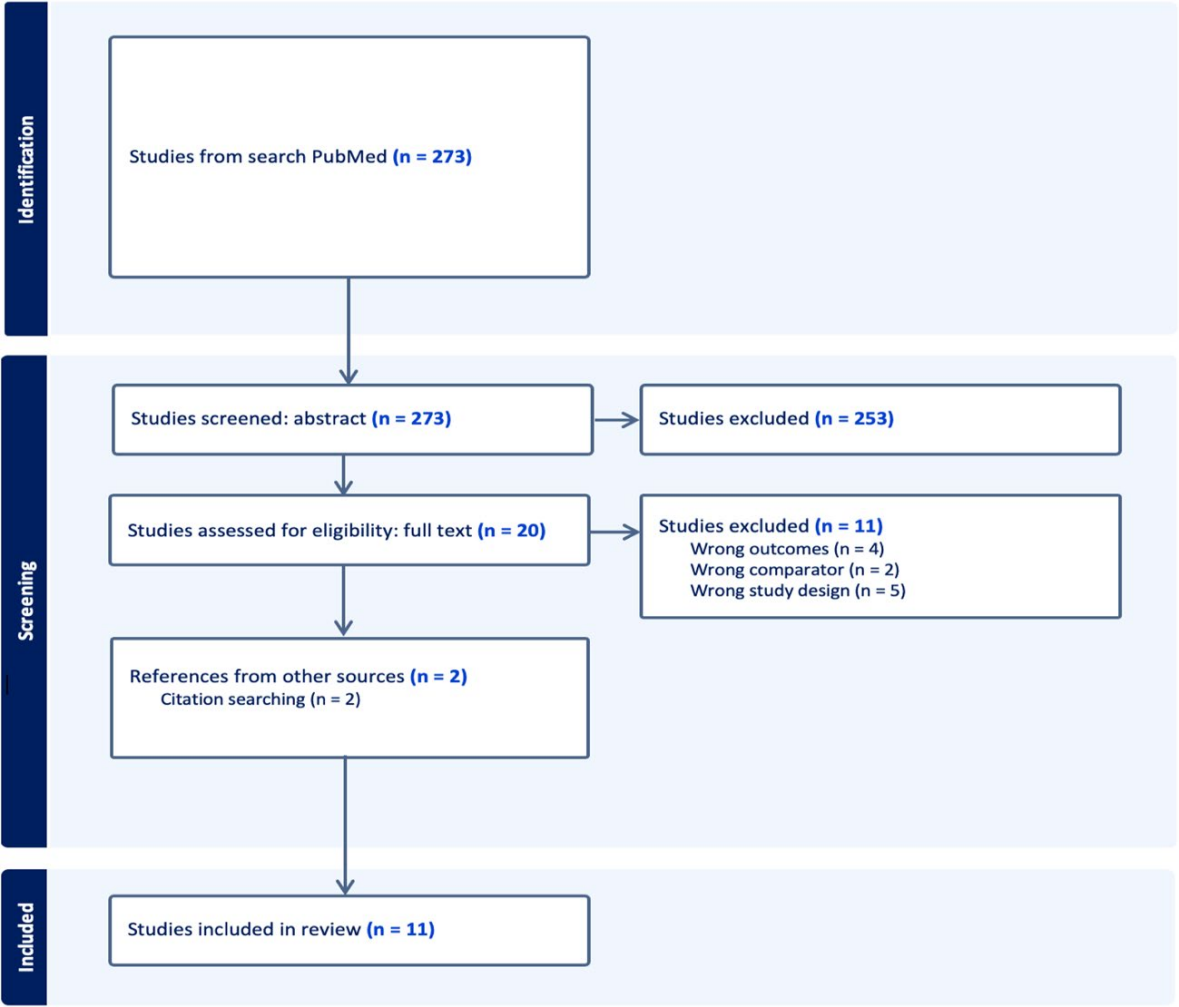
Illustrates the search results obtained from the database, presented in a flowchart format adhering to the guidelines outlined by the Preferred Reporting Items for Systematic Reviews and Meta-Analyses (PRISMA)

From an initial search, 273 records were identified, and through a screening process based on pre-established criteria, this was narrowed down to 9 articles. Additionally, two more articles were discovered through citation searching. Thus, a total of 11 articles were included in this study (Fig. 1).

## Discussion

Our goal was to shed light on the array of contrast agents employed to enhance contrast in micro-CT images of (micro)vascular structures. We emphasize the importance of selecting the most suitable contrast agent based on specific research objectives and desired anatomical visualization. The comprehensive analysis of diverse contrast agents for micro-CT imaging presented in this review unveils a rich tapestry of strengths, challenges, and nuanced considerations, laying the groundwork for a thorough discussion that navigates the intricacies of agent selection, imaging outcomes, and the evolving landscape of (micro)vascular imaging research.

Some of the recently developed contrast agents have not been included into this review due to the lack of the published comparative studies. For example, µAangiofil, which enabled promising microvascular imaging in some of the preclinical animal models [18, 35]) and has the potential to become a contrast of choice for some of the small lab animal studies despite its actually rather high price.

To provide clarity and structure, this discussion session is divided following the application methods of contrast agents.

## Application method: both intravascular and through immersion

### Iodine based contrast agents

Several articles included in this research compared Fenestra VC^®^ (iodinated nano-emulsion) with Imeron-300^®^ (C_17_H_22_I_3_N_3_O_8_, iomeprol), Isovue-370^®^ (iopamidol, containing iodine), and ExiTron nano 12000^®^ (metal-based contrast agent, containing iodine) as contrast agents.

Schambach et al. [36] conducted an *in-vivo* comparison of Fenestra VC^®^ and Imeron-300^®^ in mice. Fenestra VC^®^ provided better contrast in the thoracic vasculature. In the abdominal vasculature, Fenestra VC^®^ offered clear visibility of venous vessels but had less pronounced enhancement of kidney and spleen tissue compared to Imeron-300^®^. One topic of discussion in this article was the administration of relatively large volumes of contrast agent (a bolus technique was used injecting several volumes of 200 µl Imeron-300^®^ with a flow rate of 525 µl/min) prior to, or during the *in-vivo* micro-CT procedure. This practice can potentially result in vessel widening and compromise the reliability of the data collected. The study's overarching conclusion supports the feasibility of achieving high-resolution imaging of vascular structures in small rodents, specifically mice, through *in-vivo* micro-CT angiography.

Lalwani et al. [23] compared Fenestra VC^®^ with Isovue-370^®^ (iopamidol, containing iodine) and ExiTron nano 12000^®^ (metal-based contrast agent, containing iodine) in the thoracic region of *in-vivo* mice, including lungs and heart, especially focusing on (micro)vasculature and quantitative evaluation of lung tumors. ExiTron nano 12000^®^ provided the highest contrast enhancement, and the most distinct demarcation of tumor margins was observed. The iodine concentration varied significantly among the three contrast agents examined. ExiTron nano 12000^®^ exhibited the advantage of requiring a small volume (100µL) to achieve the required level of contrast. Fenestra VC^®^ had a lower iodine concentration of 50 mg/ mL, leading to suboptimal contrast enhancement compared to ExiTron nano 12000^®^. However, the authors were unable to explore the use of a higher iodine concentration due to institutional IACUC guidelines on intravenous dosing limits, which would have been surpassed by the necessary volume. As a result, optimal staining was not achieved.

Both above-described studies underscore the importance of selecting contrast agents based on specific imaging goals and highlight challenges related to contrast agent characteristics, administration protocols, and institutional dosing limits in living small rodent imaging.

**Fig. 2:**
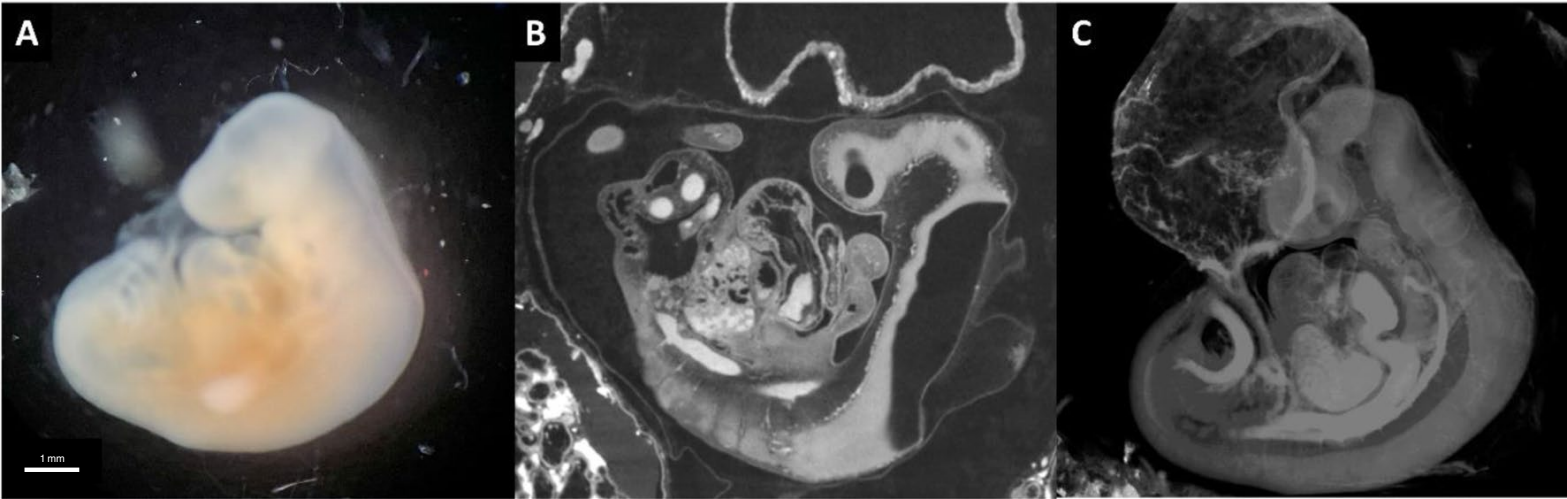
Human embryo sample 1, Carnegie Stage 14, corresponding to gestational age of approximately 7 weeks, 6 mm in total length. **A** microscopic image of human embryo (image taken after dissection from fallopian tube). **B** Micro-CT image at approximately midsagittal level (2.5 µm voxel size), showing in white vascular structures. **C** 3D rendering with Bruker CTvox software, showing in white all vascular structures

**Fig. 3:**
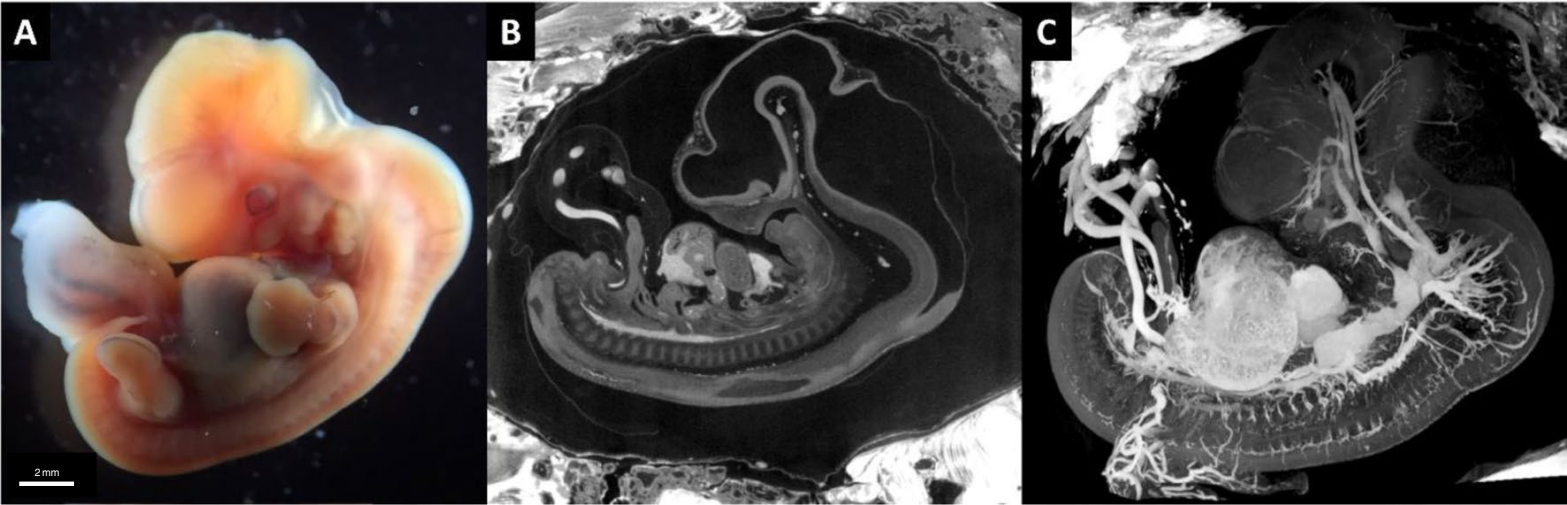
Human embryo sample 2, Carnegie Stage 18, corresponding to gestational age of approximately 8,5 weeks, 15 mm in total length. **A** microscopic image of human embryo (image taken after dissection from fallopian tube). **B** Micro-CT image at approximately midsagittal level (9 µm voxel size), showing in white are vascular structures. **C** 3D rendering with Bruker CTvox software, showing in white all vascular structures

To Summarize, in these comparative studies, Iodine-based contrast agents, such as ExiTron nano 12000^®^ and Imeron-300^®^, have demonstrated varying efficacy in enhancing imaging quality, with considerations including contrast enhancement levels, tumor delineation, and iodine concentration, which may be influenced by factors like administration volumes and institutional dosing limits. The osmolarity of diluted solution is 308 mOsm/L, which is equal to the osmolality of saline [6, 10]. This highlights the feasibility of using Iodine based solution as a contrast agent for biological imaging applications. In addition,

When aiming to visualize cranio-facial vascular development during early embryonic stages, it's important to recognize that vascular structures are still undergoing development and do not yet exhibit vascular continuity. Consequently, intravascular contrast agents are not applicable. Instead, immersion techniques should be employed. Dawood et al. have demonstrated that an iodine-based contrast agent, buffered Lugol (B-Lugol), yields excellent results in this regard. To the best of our knowledge, no comparative studies were performed comparing (Buffered) Lugol’s solution to other contrast agents. In addition to this scoping review, we conducted an experimental study aiming at visualization of (micro)vasculature of human embryos at an early stage of development. We acquired two human embryos (Carnegie Stages 14 and 18, corresponding to gestational ages of approximately 7 weeks and 8.5 weeks, respectively) from the Dutch Fetal Biobank. Both samples were obtained following surgical removal of the entire fallopian tube due to an ectopic pregnancy. After dissecting most of the fallopian tube, we immersed both sample in (Buffered) Lugol’s solution for 5 days, followed by rinsing in fresh PBS for 1 day before scanning them using the TESCAN UniTOM XL. We demonstrate the promising results of this experiment in Figs. 2 and 3. Detailed overview is provided of the (micro)vasculature of the developing human embryo at two stages of development. Future 3D reconstruction will be made using Amira or equivalent software enabling us to undertake quantitative research and optimize 3D visualization.

### Barium sulfate

The articles reviewed in this study that compared barium sulfate included comparisons with iohexol, lead oxide, and Microfil^®^ (silicon-containing lead chromate).

Barium sulfate consistently emerges as the preferred contrast agent for vascular imaging in diverse anatomical regions and animal models, indicated by multiple comparative studies. Xu et al. (2016) compared barium sulfate to iohexol in rabbits and, despite the larger size of rabbits and less intricate vasculature, barium sulfate proved superior, due to iohexol's rapid elimination. However, the study acknowledges limitations, such as a minimum field of view and the unattainability of repeated measurements, which may impact the accuracy of 3D models.

**Fig. 4:**
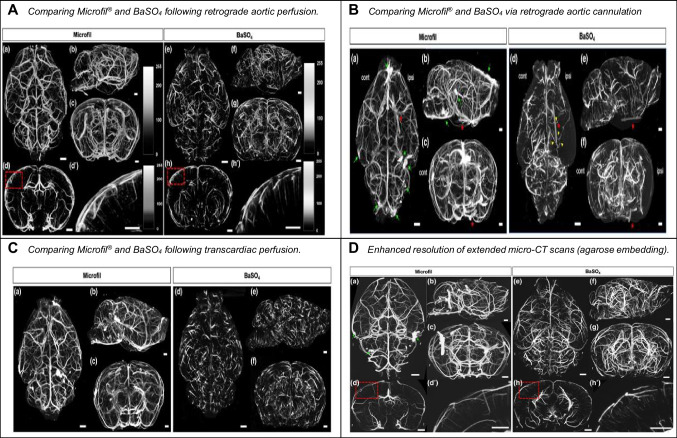
Demonstrates the differences of the ability of vascular casting, they perfused mice with Microfil or BaSO4. A-D shows different micro-CT images from the adult mouse brain. **A** Comparing Microfil^®^ and BaSO_4_ following retrograde aortic perfusion. **B** Comparing Microfil^®^ and BaSO_4_ via retrograde aortic cannulation. **C** Comparing Microfil.^®^ and BaSO_4_ following transcardiac perfusion. **D** Enhanced resolution of extended micro-CT scans through agarose embedding. Published with permission from Hong et al. (2019)

Further comparative studies by Roche et al. [33], Hong et al. (2019), and Liu et al. [26] emphasize barium sulfate's advantages over Microfil^®^ and lead oxide. Roche et al. [33] highlight barium sulfate's cost-effectiveness and convenience for bone vascular quantification, contrasting with Microfil^®^'s limitations in quantifying bone vascular networks. Hong et al. (2019) suggest barium sulfate's success in low-viscosity filling of circulations, cautioning against Microfil^®^ due to the risk of vascular complications. A positive characteristic of Microfil^®^, making it well-suited for *ex-vivo* vascular imaging, is its low shrinkage [12] (Fig. 4).

Liu et al. [26] reinforce barium sulfate's superiority over lead oxide for perfusion imaging and 3D reconstruction of micro vessels in the cervical spinal cord. Barium sulfate earns praise for high resolution, cost-effectiveness, and non-toxic properties, while lead oxide faces criticism for metal artifacts and toxicity.

Collectively, these studies position barium sulfate as the premier choice for micro-CT imaging, especially in vascular applications, offering benefits in terms of efficacy, cost-effectiveness, and safety compared to alternative contrast agents.

### Application method: intravascular (injection)

#### Gold nanoparticles

In a study conducted by Nebuloni et al. [28], AuroVist 15 nm^®^—containing gold nanoparticles—was compared with ExiTron nano 12000^®^, eXIA 160XL^®^, and Iomeron-400^®^, across various imaging applications. For this *in-vivo* mice study, the authors used a mouse holder specifically designed for vascular imaging with micro-CT and granted access via the lateral caudal veins for contrast injection. The study's key findings reveal that AuroVist 15 nm^®^ exhibited superior contrast, attributed to the highly absorbing properties of gold nanoparticles. AuroVist 15 nm^®^ will finally be eliminated via the liver and spleen, which makes it also suitable for the imaging of these organs, as well as the vasculature itself [28]. Additionally, due to this delayed clearance of gold nanoparticles, blood vessels can still be anatomically represented 2 to 3 days post-injection. On the other hand, ExiTron nano 12000^®^ showed promise for extended imaging times but required recovery intervals between injections due to reduced clearance rates observed after multiple administrations.

AuroVist 15 nm^®^ was deemed optimal for anatomical investigations of the vascular network, showcasing its suitability for such imaging applications. Iomeron-400^®^ displayed high-attenuation characteristics, yet its contrast properties were relatively low, possibly owing to contrast leakage into the extravascular compartment. Additionally, ExiTron nano 12000^®^ exhibited potential for longitudinal monitoring, suggesting its effectiveness through repeated injections for continuous imaging assessments.

The study underscores the importance of adhering to recommended dosing guidelines and administration techniques for contrast agents to ensure safety and effective imaging. In summary, AuroVist 15 nm^®^ emerges as a promising choice for anatomical investigations of the vascular network, particularly for sustained blood vessel visualization. Iomeron-400^®^ may find application in perfusion analysis, while ExiTron nano 12000^®^ holds promise for longitudinal monitoring, emphasizing the significance of following proper dosing and administration protocols. These findings contribute valuable insights for selecting contrast agents tailored to specific imaging needs and applications.

### PTA

In the study conducted by Doost and Arnolda [8], the comparison between phosphotungstic acid (PTA) and potassium iodide (iodine) as contrast agents for micro-CT imaging yielded notable findings. Iodine-stained soft tissue proved to be a non-destructive and efficient method for visualizing animal heart models, ensuring accuracy in the analysis. Notably, iodine exhibited superior efficacy over PTA in achieving complete myocardial staining in mice, a distinction attributed to iodine's smaller molecular size and differences in polarity. The advantageous small size of iodine, approximately 20 times smaller than PTA, allowed for more rapid tissue penetration, contributing to reduced staining time. The study adopted an embedding approach for contrast agent application, aiming to minimize potential damage to vascular structures compared to intravenous injections. However, the authors acknowledged that the quality of visualization achieved through embedding might not be directly comparable to the injection-based method.

In conclusion, the study suggests that iodine stands out as a more efficient contrast agent than PTA for micro-CT imaging, especially in achieving rapid and complete *ex-vivo* myocardial staining in mice. The choice between immersing and intravenous injections should be considered based on the specific imaging goals, acknowledging potential differences in visualization quality.

**Fig. 5:**
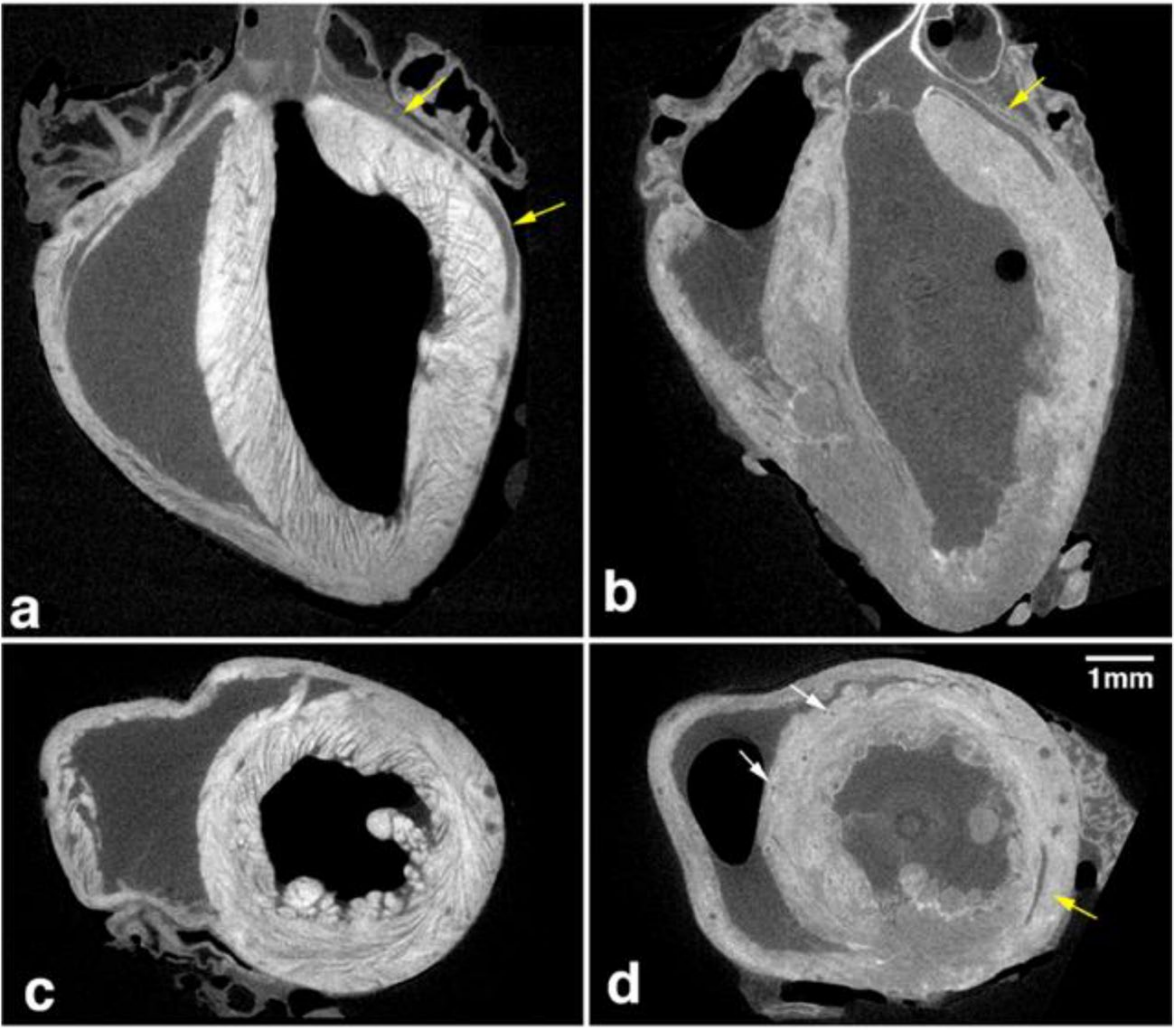
coronal **a**, **b** and axial **c**, **d** sections extracted from micro-CT images of the mouse heart, obtained from a mouse infused with iodine (Lugol’s solution) (a, c) or PTA (b, d). Published with permission, Dunmore-Buyze et al. [10]

In their 2014 study, Dunmore-Buyze et al. conducted a comparison between iodine (Lugol's solution) and PTA as contrast agents for micro-CT imaging. Lugol's solution, containing iodine, demonstrated robust and uniform enhancement of ventricular (Fig. 5) and bronchiolar walls. In contrast, PTA staining resulted in weaker ventricular myocardial enhancement and lacked distinction in bronchial walls. Both methods exhibited contrast uptake in the renal cortex and stomach lining, with PTA-infused mice showing higher uptake levels. Iodine, notably Lugol's solution, proves advantageous for comprehensive myocardial staining and provides sufficient contrast for the renal cortex and stomach lining. Conversely, PTA staining may be preferable for its robust enhancement of liver parenchyma.

In the investigations performed by Doost and Arnolda [8] and Dunmore-Buyze et al. [10], both observed that, compared to PTA, iodine (Lugol's solution and potassium iodide) has a superior efficacy in achieving comprehensive myocardial staining in mice, which was attributed to iodine's smaller molecular size and distinct polarity. While iodine, particularly Lugol's solution, provided strong and uniform enhancement of ventricular walls and bronchiolar walls, PTA staining resulted in weaker ventricular myocardial enhancement emphasizing the importance of selecting the appropriate contrast agent based on specific research objectives and desired anatomical visualization.

### Summary

The above-described comparative studies evaluating contrast agents for micro-CT, all focused on imaging of (micro)vascular structures and showed that barium sulfate consistently emerges as the preferred choice for vascular applications, demonstrating efficacy, cost-effectiveness, and safety benefits over alternatives like iohexol, lead oxide, and Microfil^®^. Iodine-based contrast agents, including Fenestra VC^®^, Imeron-300^®^, Isovue-370^®^, ExiTron nano 12000^®^ and Lugol’s solution, exhibit varying efficacy levels in enhancing imaging quality, emphasizing considerations such as contrast enhancement, tumor delineation, and iodine concentration, influenced by factors like administration volumes and dosing limits. Samples immersed for specific periods of time, like (Buffered) Lugol’s solution, show promises for obtaining high quality images of (micro)vasculature of human embryos. Additionally, gold nanoparticle-based AuroVist 15 nm^®^ shows promise for vascular network investigations, while phosphotungstic acid (PTA) and potassium iodide (iodine) comparisons suggest iodine's superiority in achieving comprehensive myocardial staining, with implications for immersing versus intravenous injection methods. Aiming in visualization of early cranio-facial vascular development, intravascular contrast agents are unsuitable due to ongoing developmental processes and lack of vascular continuity. Immersion techniques, particularly utilizing buffered Lugol (B-Lugol), have been shown effective by Dawood et al. A summary of specific characteristics is provided in Table 3.

**Table 3:** Presenting specific characteristics of the incorporated contrast agents

Contrast agent	Atomic number	Chemical formula	Toxicity	prices	Intravascular / immersing / both	*Ex-vivo* / *In-vivo* / both	Mirco-CT study aim
Barium Sulfate	56	BaSO_4_	+	€	Intravascular	oth	Particularly preferred for vascular visualization
Gold nanoparticles	79	AU (aurum)	+	€€€	Intravascular	Both	Superior contrast due to gold nanoparticles; potential for prolonged visualization
Iodine	53	(several, depending on combined characteristics)	+ +	€	Both	oth	Often used for vascular imaging and soft tissue visualization
Lead Oxide	82	Pb_3_O_4_	+ + +	€	Intravascular	*x-vivo*	Particularly suitable for vascular imaging
Microfil	82 (lead)	Pb_3_O_4_/Silicone	+ + +	€€	Intravascular	*Ex-vivo*	High, often used for vascular casting and imaging
Phospotungistic acid (PTA)	74	H_3_PW_12_O_40_	+ + +	€€	Intravascular	*Ex-vivo*	Variable; preferred for strong liver parenchymal enhancement
PU4ii	varies	polyurethane-based resin	+	€€	Intravascular	*Ex-vivo*	Variable; characterized by hydrophobic properties

- Toxicity: +  = low / +  +  = moderate / +  +  +  = severe

- Price: € = low / €€ = moderate / €€€ = high

### Limitations

While our research was extensive and utilized the PubMed database to gather pertinent literature for this review, it is important to recognize that not all existing contrast agents were encompassed. Our focus was primarily on those examined in comparative studies, potentially overlooking other commonly used or well-known contrast agents. To provide a comprehensive overview of available options, further investigation is warranted to evaluate and compare the outcomes of these excluded contrast agents. Furthermore, we urge researchers to delve into the exploration of novel or modified contrast agents to continuously advance the search for the most optimal options in (micro)vascular imaging.

## Conclusion

Our goal was to shed light on the array of contrast agents employed to enhance contrast in micro-CT images of (micro)vascular structures. We emphasize the importance of selecting the most suitable contrast agent based on specific research objectives and desired anatomical visualization.

When aiming for optimal vascular imaging, crucial factors revolve around the choice between *in-vivo* or *ex-vivo* application, as well as the distinction between purely vascular and combined vascular imaging with other hard or soft tissue structures. In scenarios where the focus is solely on vascular visualization, intra-arterial contrast agents may be the optimal choice. Conversely, when aiming to visualize vasculature in relation to surrounding tissue structures, immersing application techniques are often recommended. In both in vivo and ex vivo settings, promising options for contrast agents include barium sulfate and iodine-based agents, such as buffered Lugol, potassium iodide, and XlinCA^®^. Iodine's smaller molecular size enables rapid tissue penetration, leading to superior efficacy in achieving thorough staining and efficient soft tissue visualization. XlinCA^®^ offers potential advantages over plastic resin-based PU4ii^®^, including ease of use, more reliable filling of anatomical structures, and simultaneous perfusion of multiple regions in a single procedure. Barium sulfate consistently proves to be a preferred choice across various anatomical regions due to its efficacy, cost-effectiveness, and safety benefits compared to alternatives. However, each contrast agent has its own advantages and challenges. Phosphotungstic acid (PTA) may be preferred for robust liver parenchymal enhancement, while considerations for soft tissue visualization and staining times often favor iodine-based agents. Additionally, we highlight the potential of (buffered) Lugol solution, demonstrated through our visualization of (micro)vascular structures in human embryonic samples in an ex vivo setting. The choice between embedding animals and utilizing intravenous injections entails a trade-off between the risk of potential vascular damage and the quality of visualization.

Whit this scoping review we contribute to the understanding of contrast agents in micro-CT imaging. It is essential to note the need for careful consideration of protocols, imaging techniques, and the specific characteristics of the tissues under investigation. Future research should continue to explore innovative contrast agents and refining imaging methodologies to further enhance the accuracy, efficiency, and applicability of micro-CT imaging in both research and clinical settings.

## Data Availability

The data that support the findings of this study are available from the corresponding author, K.J., upon reasonable request.

## Appendix 1

Characteristics of included contrast agents and its application method

### Barium sulfate

Barium sulfate is an inorganic, radiopaque contrast agent characterized by a relatively high atomic number (56), resulting in efficient absorption of radiation. Barium sulfate is regarded as relatively safe as a contrast agent, primarily due to its inert nature and limited bioavailability through absorption and metabolism within the human body. Typically, barium sulfate is formulated as a suspended medium to achieve optimal consistency and facilitate dispersion within the intricate vasculature [22], Xu et al., 2016).

### Iodine

Iodine is commonly used as contrast agent. This contrast medium typically exhibit higher viscosity and greater osmolality (higher concentration of solute molecules per kilogram of water) compared to blood or plasma. The osmolality levels vary among different iodine contrast agents. Iodine contrast agents can be classified into two categories: ionic and non-ionic contrast agents. Ionic contrast agents consist of molecules that carry positive or negative charges, while non-ionic contrast agents do not possess any electric charge. Non-ionic contrast agents are more commonly used (*in-vivo* and *ex-vivo*) today because they are generally considered safer and have a lower risk of adverse reactions, like anaphylactoid reactions (*in-vivo*), compared to ionic contrast agents [37]. Commonly employed Iodine based contrast agents (commercial or self-made products) with their own specific characteristics are presented in the list below.

- Iomeron^®^/Imeron^®^ contains Iomeprol (C_17_H_22_I_3_N_3_O_8_), a non-ionic, low-osmolar, water-soluble contrast agent [[39].
- Omnipaque^®^ contains Iohexol (C_19_H_26_I_3_N_3_O_9_), which is a non-ionic, low-osmolar, water-soluble contrast agent [[29].
- Isovue^®^ contains Iopamidol, a non-ionic, water-soluble contrast agent with low-osmalilty [[2].
- eXIA is a blood pool contrast agent characterized by its aqueous iodine dispersion [1].
- Fenestra VC^®^ is an iodinated nano-emulsion. The lipid emulsion particles undergo surface modification with DSPE-PEG, leading to an extended circulation time of the contrast agent [[16].
- XlinCA is a water-soluble contrast agent that possesses a substantial molecular weight, low viscosity, and a high concentration of iodine.
- ExiTron nano 12000^®^is a commercial alkaline earth metal-based contrast agent, for preclinical use. It is inclined to enhance the visualization of the vasculature [[27].
- Lugol’s solution (I_3_K): Lugol's solution is prepared by combining aqueous potassium iodide with iodine. B-Lugol, is the buffered form of Lugol.
- Potassium iodide (I_2_kI): potassium iodide is similar to Lugol's solution, but with a 90% ethanol formulation instead of the aqueous solution.

### Lead

Lead, e.g. as lead oxide, has been used in some research settings, however, it's important to highlight the potential risks associated with its toxicity. Researchers and practitioners must adhere to strict safety guidelines and consider alternative contrast agents when possible to minimize exposure to harmful substances. Commercially, Microfil^®^ is available, which is a radio-opaque silicone rubber that contains particles of lead chromate and lead sulfate.

### PU4ii^®^

PU4ii^®^ is a hydrophobic polyurethane-based resin that exhibits limited permeability across the hydrated endothelial cell layer and undergoes polymerization upon injection [24]. In addition to its application in Micro-CT tomography, PU4ii^®^ is frequently utilized as a contrast agent for various scanning modalities, including the Scanning Electron Microscope.

### Gold nanoparticles

Gold nanoparticles (AuNPs) used for contrast agents are inorganic. Gold demonstrates a pronounced X-ray attenuation coefficient and an extended vascular retention time in comparison to alternative contrast agents. AuNPs are highly appealing due to their potential for excellent biocompatibility and can undergo surface functionalization for the purpose of targeted delivery [3]. There are two commercially available agents, namely, Aurovist 15 nm^®^ a blood pool contrast agent, consisting of gold nanoparticles with a diameter of 15 nm and ExiTron nano 12000^®^ (a metal-based contrast agent, containing also iodine) [28].

### Phospotungstic acid (PTA)

Phosphotungstic acid (PTA) serves as a stain with an affinity for proteins, particularly collagen. Classified as a relatively sizable inorganic polar molecule, PTA is utilized as dilution in water. Tungsten, alternatively known as wolfram, contributes to PTA's effectiveness by possessing a high atomic number, thereby ensuring heightened contrast, as highlighted in the research article by Degenhardt et al. [6]. The ability of PTA to selectively bind to proteins, especially collagen, makes it a valuable contrast agent for various imaging techniques, with its application extending beyond Micro-CT tomography to include modalities like the Scanning Electron Microscope.
